# Widespread Cutaneous Metastases as the Primary Presentation of an Occult Neuroendocrine Tumour of Unknown Primary

**DOI:** 10.7759/cureus.116378

**Published:** 2026-09-16

**Authors:** Anesa Noor, Fatma Shah, Navdeep Sodhi, Salih Bakhiet

**Affiliations:** 1 Internal Medicine, Northampton General Hospital, Northampton, GBR; 2 Pathology, Northampton General Hospital, Northampton, GBR

**Keywords:** histopathology, immunohistochemistry (ihc), metastatic cancer of unknown primary, metastatic neuroendocrine tumor, neuroendocrine neoplasm

## Abstract

Cutaneous metastases from neuroendocrine neoplasms (NENs) are extremely rare even in advanced disease and may rarely be the initial presentation of an occult primary tumour. We describe a woman in her late 30s who presented with slowly growing skin lesions over a period of two years without associated systemic symptoms. She later developed abdominal discomfort, prompting hospital assessment. Clinical examination revealed extensive cutaneous nodules and a palpable abdominal mass. Imaging demonstrated widespread metastatic disease with multiple soft tissue deposits involving the liver, brain, and other deep organs. Histopathological examination of skin biopsies confirmed a grade 3 metastatic neuroendocrine tumour (NET) with a possible bronchial primary origin. The patient received systemic chemotherapy in addition to palliative radiotherapy for symptomatic lesions. Despite treatment, her condition progressively deteriorated due to the extensive disease burden, and management was subsequently redirected towards best supportive care with a focus on symptom control. This case highlights the diagnostic challenge posed by the variable cutaneous presentations of NENs, which can often be mistaken for benign growths without a high index of suspicion. This emphasises the importance of early recognition and biopsy of suspicious skin lesions.

## Introduction

Neuroendocrine neoplasms (NENs) represent a heterogeneous group of tumours derived from neuroendocrine cells, which may arise from any organ [1]. Their prevalence corresponds to the concentration of neuroendocrine cells, making the gastrointestinal tract and pancreas the most common primary sites of origin reported (approximately 70%), followed by the lungs (approximately 30%), along with several other rare sites described, including cases presenting with metastases of unknown primary in approximately 20% of patients [2,3].

NENs, previously considered uncommon, have demonstrated a significant increase in incidence over recent decades, with a reported fourfold increase in England between 1995 and 2018; however, it remains unclear whether this increase is due to improved detection methods or a true rise in tumour incidence [4].

According to the 2022 World Health Organization (WHO) classification, NENs are histologically divided into two main categories: well-differentiated neuroendocrine tumours (NETs) and poorly differentiated neuroendocrine carcinomas (NECs). NETs are further graded based on cellular proliferative activity as determined by the mitotic count and Kiel 67 (Ki-67) index [5].

The clinical presentation of NENs varies widely depending on their secretory activity. The majority are nonfunctioning tumours with no specific hormonal activity and therefore are often detected at advanced stages. Functioning NENs can produce a wide spectrum of endocrine-related symptoms depending on the hormone secreted, such as carcinoid syndrome, insulinoma, gastrinoma, vasoactive intestinal peptide-secreting tumours (VIPoma), glucagonoma, and somatostatinoma [6].

Cutaneous metastases from solid malignancies are rare, with an estimated incidence of less than 10%. They most commonly arise from breast, renal, ovarian, bladder, lung, colorectal and prostate primaries, as well as melanomas. The clinical presentation is highly variable and may mimic inflammatory conditions, benign growths or primary skin neoplasms, making diagnosis challenging without a high index of suspicion [7].

## Case presentation

A woman in her late 30s was advised by her primary care physician to attend the same-day emergency care unit for assessment after she presented with a three-day history of worsening abdominal discomfort and was noted to have a palpable mass in her abdomen. Her past medical history was unremarkable apart from undergoing three elective caesarean sections resulting in live births of healthy children. She had no significant chronic medical conditions and was on no regular medication.

On assessment, her vital signs were within normal limits, and she was afebrile. Examination revealed numerous widespread cutaneous nodules involving the forehead, neck, back, breasts, trunk, abdomen, and thighs (Figure 1). Although these nodules were mostly painless, a deep, irregular, firm mass was palpable at the site of her abdominal pain. The lesions ranged in size from approximately 1 cm to a breast lesion measuring 8 x 5 cm. These nodules had varied characteristics; some showed superficial scarring and oozing, some were hard and fixed with no clear edges, while others were mobile.

**Figure 1:**
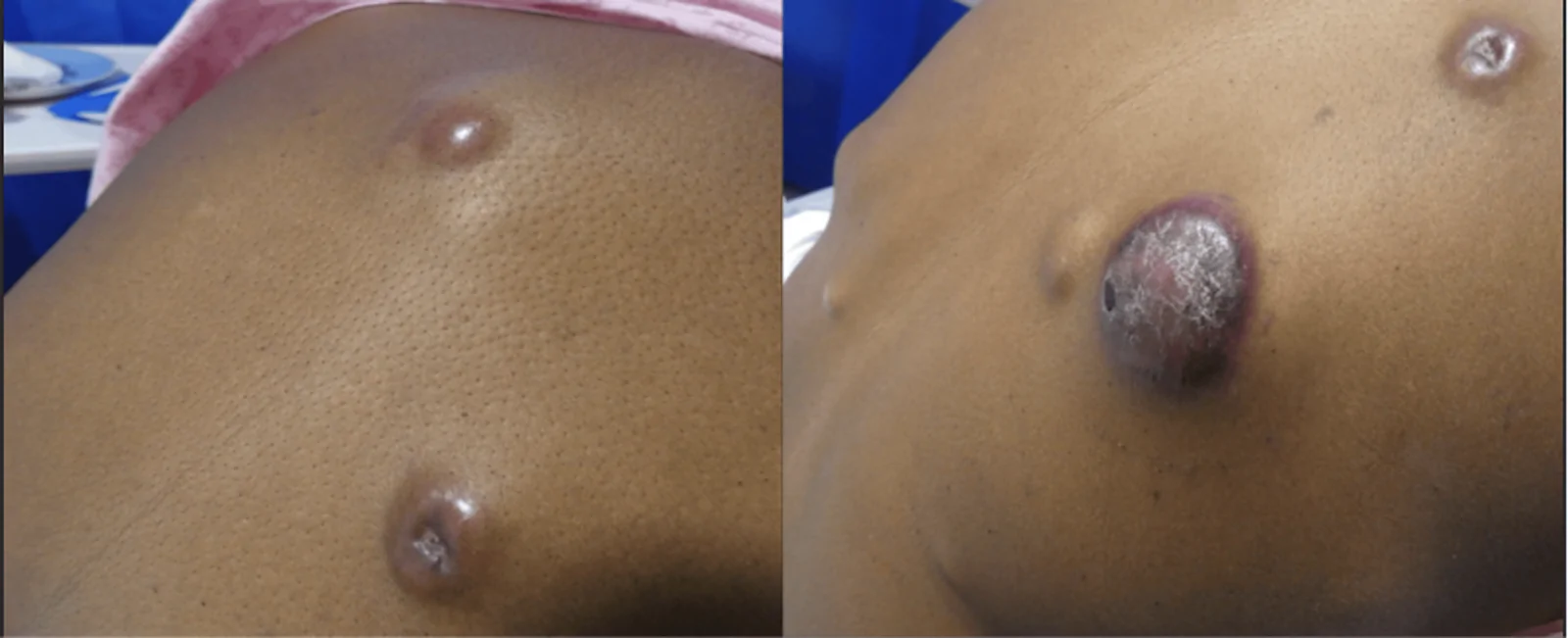
Clinical photographs demonstrating multiple cutaneous and subcutaneous lesions of varying sizes and morphology involving the upper back with several lesions demonstrate surface ulceration and scaring.

Further detailed history revealed that the patient had been developing these lesions gradually over the past two years after the delivery of her youngest child. The first lesion developed over her right thigh, which was investigated in primary care with ultrasonography describing it as a small soft tissue growth of no clinical significance. Therefore, this was thought to likely be a sebaceous cyst and a routine referral to the dermatology clinic was made; however, she had not yet been seen at the time of presentation. The patient also reported significant unintentional weight loss over the same period (approximately 20% of her total body weight) without associated systemic symptoms, such as night sweats, flushing, altered bowel habits, chronic cough, or headaches.

Investigations

Initial blood results were unremarkable apart from a mild microcytosis (mean corpuscular volume of 80 fL) and iron deficiency (iron level of 2 μmol/L and transferrin saturation of 4%) with a normal haemoglobin level of 126 g/L. Lactate dehydrogenase (LDH) was mildly raised at 224 IU/L (normal range 135-214 IU/L). Otherwise, renal and liver function tests, electrolytes, and inflammatory markers were all normal. A blood-borne viral screen for human immunodeficiency virus (HIV), hepatitis B, and hepatitis C was negative.

She underwent a contrast-enhanced computed tomography (CT) scan of the abdomen, which revealed multiple large lobulated soft tissue density lesions involving the liver, retroperitoneum, and abdominal wall (Figure 2). The scan also showed proximal portal and splenic vein thrombosis with secondary portosystemic shunts/collaterals, findings all suggestive of metastatic disease of an unclear primary. Given the concerning imaging findings, a whole-body contrast-enhanced CT scan was performed, which showed multiple enhancing cortical lesions in both cerebral hemispheres without significant oedema or mass effect. There were numerous bulky nodal and soft tissue deposits in the lower neck involving the right lobe of the thyroid gland and left parotid gland, as well as the anterior and posterior chest wall involving both breasts.

**Figure 2:**
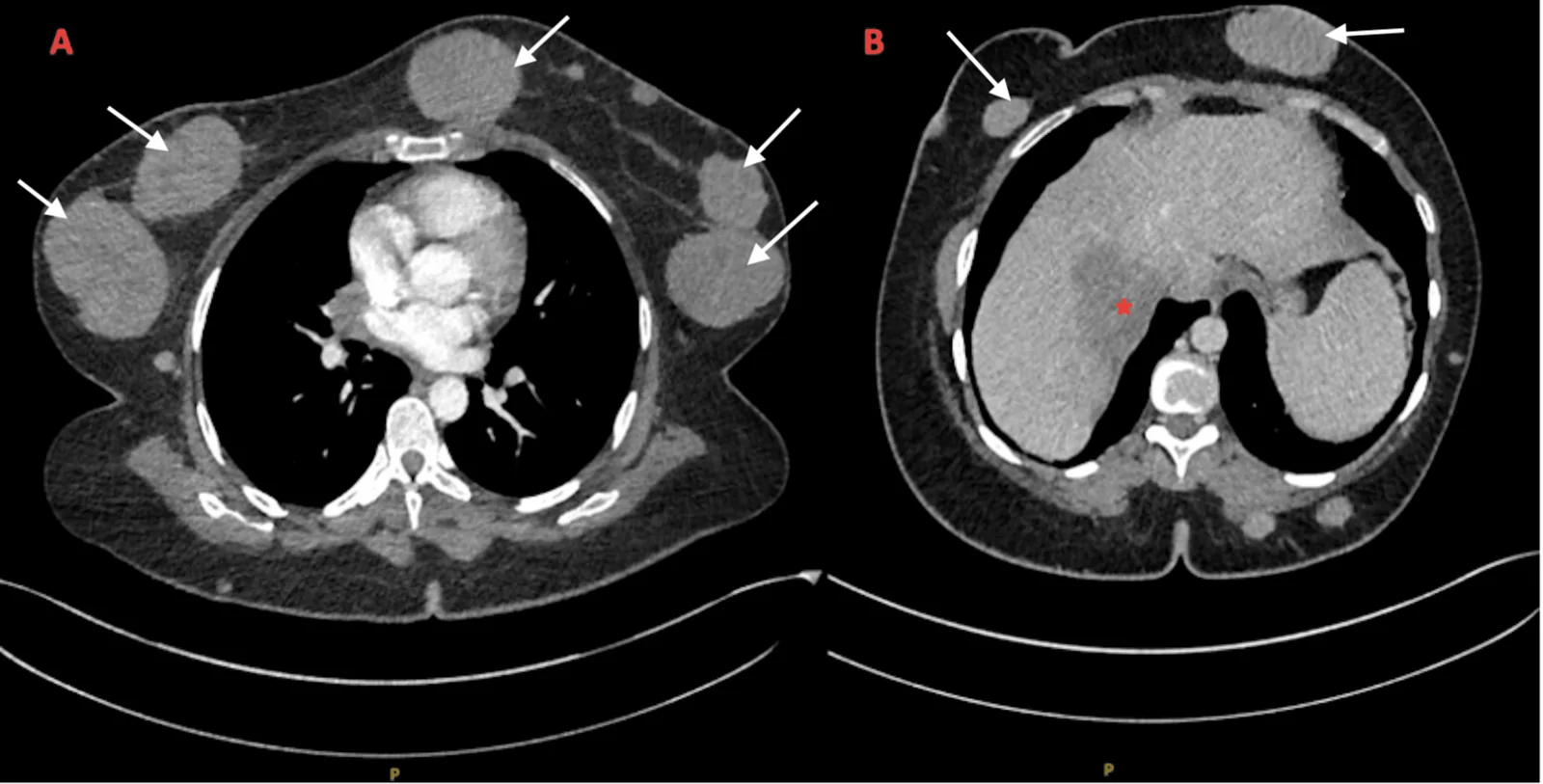
Cross sectional contrast-enhanced CT images of the chest and abdomen (A) At the level of the heart showing multiple lobulated soft tissue density deposits in the chest wall. (B) At the level of the liver similarly showing deposits in the abdominal wall and back in addition to lobulated hypodense lesion along the under surface of right lobe of the liver (indicated by *). CT: computed tomography

Given the widespread and heterogeneous morphology of the cutaneous lesions, coupled with significant unintentional weight loss and multi-organ involvement, the differential diagnosis at this stage included metastatic carcinoma of unclear primary, metastatic soft tissue sarcoma, lymphoma and primary cutaneous malignancy including malignant melanoma and Merkel cell carcinoma. Primary or metastatic NEN was also considered; however, this was felt to be less likely in the absence of associated systemic symptoms such as flushing, altered bowel habits or palpations. Tissue characterisation was therefore crucial in establishing the underlying diagnosis.

Biopsy samples obtained from two skin lesions demonstrated infiltration of the dermis and subcutaneous fat by tumour cells arranged in nests and sheets with finely stippled chromatin suggestive of a possible neuroendocrine tumour. Immunohistochemical analysis was conducted in a stepwise manner, first with broad-spectrum cytokeratins (MNF116 and AE1/AE3), which were diffusely positive, supporting an epithelial cell lineage. SOX10, Melan-A, S100 and desmin were all negative, excluding melanoma and myogenic sarcoma. To further characterise the epithelial phenotype, a panel including PAX8 (Müllerian, thyroid or renal origin), CDX2 (enteric origin), GATA3 (breast or urothelial origin) and CD34 (gastrointestinal stromal tumour or vascular origin) was performed; these markers were also negative, ruling out metastatic carcinoma from these primary sites. 

Positivity for synaptophysin, chromogranin A and CD56 confirmed neuroendocrine origin with an initial estimated Ki-67 labelling index of 10-15% (Figure 3). Tumour cells also expressed cytokeratin (CK) 7 and TTF-1 but were CK20 negative, which ruled out Merkel cell carcinoma (Figure 4). In summary, these findings supported a diagnosis of metastatic neuroendocrine tumour of a non-cutaneous origin.

**Figure 3:**
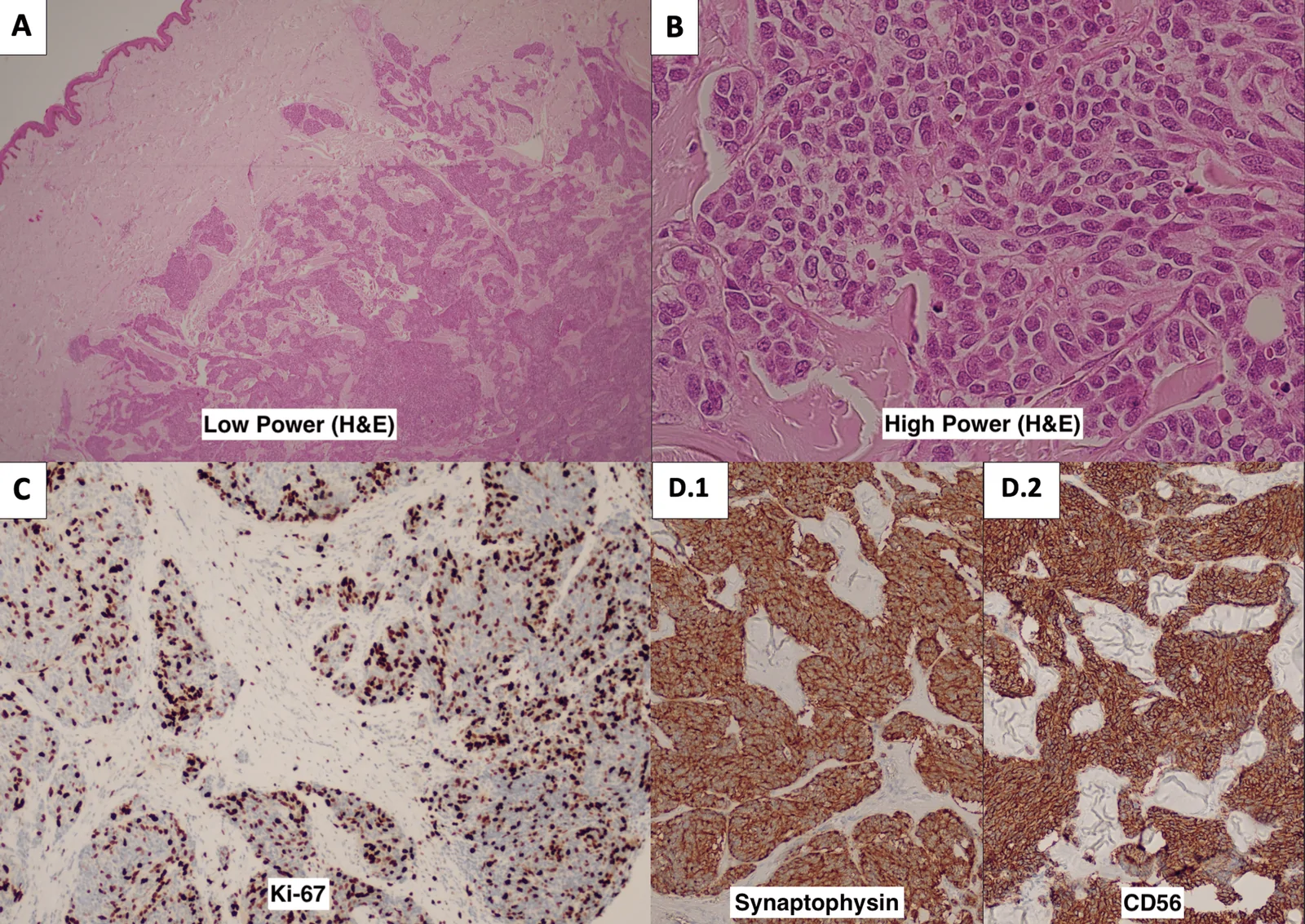
Histopathology and immunohistochemistry of the cutaneous lesions (A) Low-power microscopic examination with H&E staining showing skin with an unremarkable overlying epidermis; however, within the underlying dermis, there is an ill-defined epithelial neoplasm arranged in nests and trabeculae, separated from the epidermis by a narrow Grenz zone. (B) High-power (H&E) view demonstrating monomorphic atypical tumour cells arranged in islands and trabeculae with round to oval nuclei and finely granular “salt-and-pepper” chromatin, consistent with a neuroendocrine tumour. (C) Immunohistochemistry showed a Ki-67 index initially reported as 10-15%, later revised to 30%. (D.1-D.2) Strong diffuse positivity of both synaptophysin (D.1) and CD56 (D.2), confirming neuroendocrine differentiation. H&E: haematoxylin and eosin, Ki-67: Kiel 67, CD: cluster of differentiation

**Figure 4:**
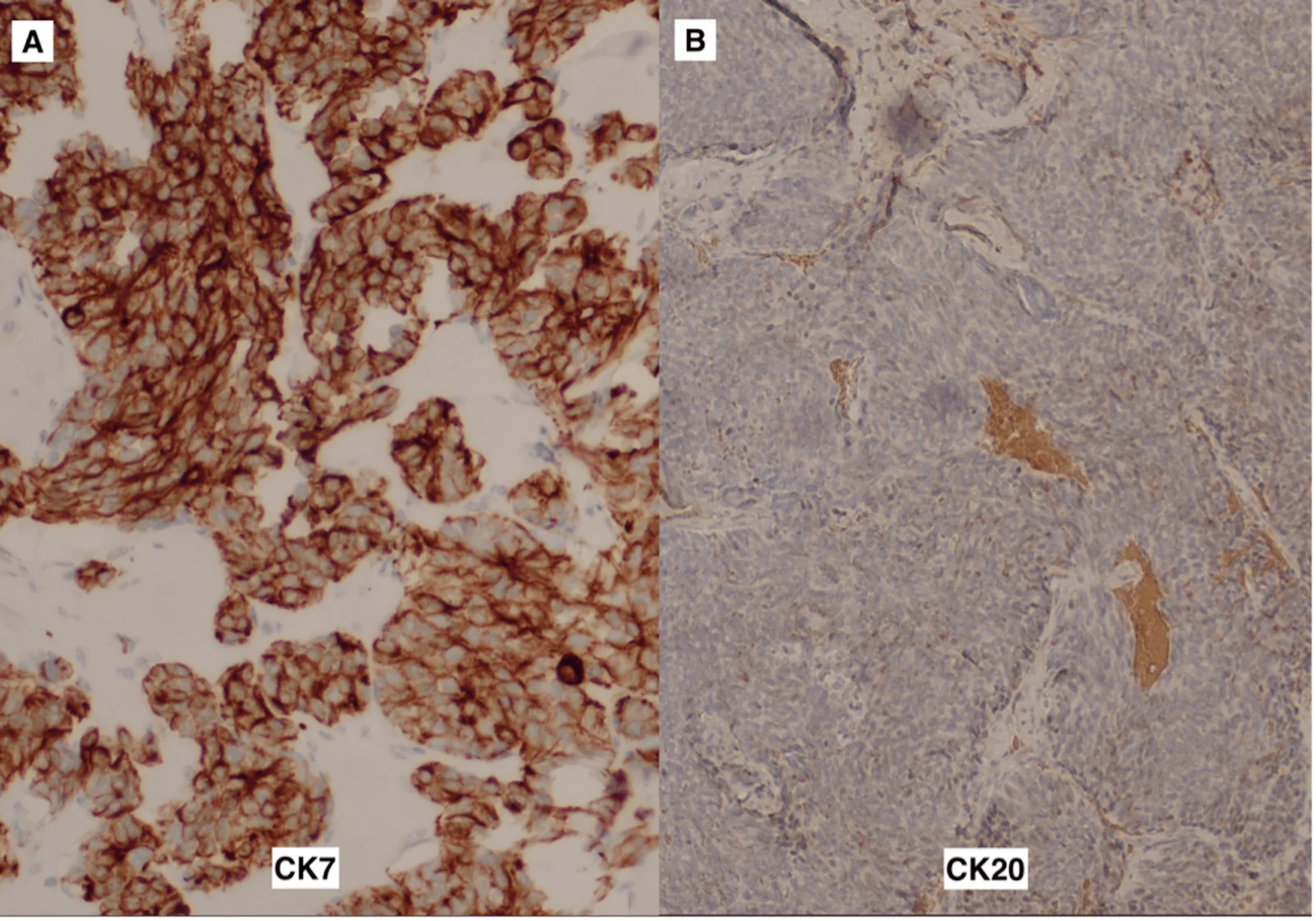
Immunohistochemistry of the cutaneous lesions Demonstrating tumour cells that are CK7 positive (A) and CK20 negative (B), which is not consistent with Merkel cell carcinoma and rather supports a diagnosis of metastatic neuroendocrine tumour of non-cutaneous origin. CK: cytokeratin

Management

The case was reviewed at the local cancer of unknown primary (CUP) multidisciplinary team meeting where the histopathology was reassessed at a tertiary centre. The Ki-67 labelling index was subsequently revised to 30%, consistent with a grade 3 metastatic neuroendocrine tumour from a possible bronchopulmonary origin given TTF-1 expression, although this marker is less specific in high-grade disease. Histological confirmation of a pulmonary primary was not obtained.

The patient subsequently disengaged from scheduled follow-up for a period of five months. Upon re-presentation, repeat imaging demonstrated significant interval disease progression with an estimated 50% increase in overall disease burden. There was extensive metastatic involvement including large mesenteric deposits with likely extension into the pancreas, in addition to metastases involving the adrenal glands, liver, lung, pleura, lymph nodes and soft tissues. Contrast-enhanced magnetic resonance imaging (MRI) of the brain showed multiple subcutaneous scalp metastases and more than fifty subcentimetre intraparenchymal supratentorial and infratentorial metastases, some with haemorrhagic changes, without significant cerebral oedema or midline shift.

Given the extensive disease burden, rapid interval progression and decline in the patient's performance status, a treatment strategy with palliative intent was adopted. This consisted of targeted radiotherapy to symptomatic subcutaneous lesions combined with systemic chemotherapy with Capecitabine and Temozolomide (CAPTEM protocol).

The patient underwent three courses of single-fraction radiotherapy (8 Gy) with good symptomatic benefit. She also completed two cycles of systemic chemotherapy; however, treatment was poorly tolerated, and a shared decision was made to discontinue chemotherapy in favour of best supportive care under specialist palliative care services.

## Discussion

Cutaneous metastases from NENs are exceptionally rare. Their recognition is clinically significant, as they almost invariably indicate disseminated disease and are associated with poor prognosis [8].

In the largest single-institution review to date, four cases of well-differentiated NETs presenting with solitary cutaneous metastases were reported, alongside a review of approximately 35 previously documented cases in the literature at that time. All patients were over 50 years of age, and the lesions were initially mistaken for benign conditions such as cysts or lipomas. Importantly, progression and mortality followed within a few years of diagnosis, highlighting the prognostic significance of such findings [9].

Further reports have broadened the clinical spectrum. One case described cutaneous NEC metastasis in which the primary site remained unknown despite exhaustive work-up [10]. Another reported a small intestinal NET with a firm cutaneous mass on the flank that developed years after the initial diagnosis, illustrating that skin involvement can arise late in the disease progression [11]. A further case described a pancreatic NEC with multiple scalp and chest nodules [12]. Giuffrida et al. reported a rare case of a young woman with grade 3 gastrointestinal NET and cutaneous metastases [13]. Finally, a 72-year-old man presenting with rapidly enlarging purplish skin nodules was ultimately diagnosed with an aggressive high-grade NEC, where biopsy confirmed neuroendocrine lineage, directing oncologic management despite uncertainty about the primary source [14].

Our case shares several features with previous reports, particularly the diagnostic challenge posed by the apparent benign skin lesions and the presence of advanced metastatic disease at presentation. However, several aspects make this presentation unusual. The patient was considerably younger than most previously reported cases, apart from the case reported by Giuffrida et al. [13]. In addition, the onset of her symptoms followed shortly after the delivery of her youngest child, and it is possible that her significant unintentional weight loss may have been attributed to physiological postpartum weight changes rather than recognised as a red flag for underlying malignancy, which may have contributed to a diagnostic delay in this context. Furthermore, the cutaneous involvement was extensive and morphologically diverse, involving the face, trunk, and limbs rather than the more commonly reported solitary and indolent lesions. The lesions evolved over a period of two years before biopsy, highlighting another potential for diagnostic delay when cutaneous metastases mimic benign dermatological conditions.

Histopathological assessment confirmed grade 3 metastatic neuroendocrine tumour with a Ki-67 index of 30%, consistent with a more aggressive tumour biology. Immunohistochemistry demonstrated a CK7-positive, CK20-negative staining profile, effectively excluding Merkel cell carcinoma. This is a critical differential diagnosis in this case, as Merkel cell carcinoma is the primary neuroendocrine carcinoma of the skin and characteristically demonstrates the inverse pattern of CK20 positivity and CK7 negativity [15]. Other common primary sites, including renal, thyroid, Müllerian, gastrointestinal, breast, and urothelial origin, had already been excluded on the basis of negative PAX8, CDX2, and GATA3 staining. TFF-1 positivity was noted, raising the possibility of bronchopulmonary origin, although this marker lacks specificity in high-grade neuroendocrine tumours and no discrete accessible pulmonary mass or lymph node was identified on initial imaging to allow histological confirmation of a primary site. In addition, given the extent of disease dissemination, further invasive investigations to establish a specific primary site were not felt to be justified as this would not have altered the patient’s subsequent management.

Despite the extensive cutaneous, visceral and intracranial metastases, the patient did not develop features of a functioning neuroendocrine syndrome. This emphasises that the absence of classic symptoms such as flushing, diarrhoea or wheeze should not exclude consideration of an advanced neuroendocrine neoplasia.

## Conclusions

This case highlights that persistent, enlarging or multiple cutaneous nodules, particularly when the lesion morphology is variable or heterogeneous, should prompt consideration of metastatic disease. Histological examination with immunohistochemistry remains essential to confirm the diagnosis and guide further investigation, especially when the primary tumour remains occult. Cutaneous metastases from NENs are rare but typically indicate advanced disseminated disease and an unfavourable prognosis. Clinicians should therefore maintain a high index of suspicion for metastatic neuroendocrine malignancy in patients with unexplained cutaneous lesions even in the absence of classical hormone-mediated syndromes.
